# Matrix Metalloproteinases -14, -9 and -2 are Localized to the Podosome and Involved in Podosome Development in the A7r5 Smooth Muscle Cell

**DOI:** 10.13188/2332-3671.1000020

**Published:** 2017-05-10

**Authors:** SE Thatcher, JE Black, H Tanaka, K Kohama, ME Fultz, LA Cassis, GL Wright

**Affiliations:** 1 Department of Pharmacology and Nutritional Sciences, University of Kentucky, USA; 2 Department of Physiology, Pharmacology and Toxicology, Marshall University, USA; 3 Department of Health Sciences, Gunma University, Japan; 4 Research Institute of Pharmaceutical Sciences, Musashino University, Japan; 5 Department of Biology and Chemistry, Morehead State University, USA

**Keywords:** Cytoskeleton, Extracellular, Degradation, Phorbol, Remodeling

## Abstract

**Aim:**

The purpose of the study was to localize matrix metalloproteinase (MMP)-14, -9, and -2 in the A7r5 smooth muscle cell and to understand the interaction between these MMPs and the cytoskeleton. This interaction was observed under non-stimulating and phorbol 12, 13-dibutyrate (PDBu)-stimulating conditions.

**Methods:**

Confocal microscopy was utilized to define the localizations of MMPs and tissue inhibitor of matrix metalloproteinases (TIMPs) in the A7r5 cell and to determine interaction between MMPs and the cytoskeleton. Under PDBu-stimulating conditions, the presence of MMP active forms and activity by gel zymography was evaluated in the A7r5 cell. Actin and microtubule-polymerization inhibitors were used to evaluate MMP interaction with the cytoskeleton and the cytoskeleton was observed on matrix and within a Type I collagen gel.

**Results:**

MMP-14, -9, and -2 were localized to the podosome in the A7r5 smooth muscle cell and interactions were seen with these MMPs and the actin cytoskeleton. PDBu-stimulation induced increases in the protein abundance of the active forms of the MMPs and MMP-2 activity was increased. MMPs also interact with a-actin and not β-tubulin in the A7r5 cell. Galardin, also known as GM-6001, was shown to inhibit podosome formation and prevented MMP localization to the podosome. This broad spectrum MMP inhibitor also prevented collagen gel contraction and prevented cell adhesion and spreading of A7r5 cells within this collagen matrix.

**Conclusion:**

MMPs are important in the formation and function of podosomes in the A7r5 smooth muscle cell. MMPs interact with a-actin and not β-tubulin in the A7r5 cell. Podosomes play an important role in cell migration and understanding the function of podosomes can lead to insights into cancer metastasis and cardiovascular disease.

## Introduction

Matrix metalloproteinases (MMPs) are endopeptidases that help to degrade extracellular components and promote vessel remodeling in the vasculature [1]. Positive or outward remodeling is caused by high levels of MMP activity resulting in a decrease in tensile strength of the vessel wall leading to such conditions as abdominal aortic aneurysms (AAAs) [2]. Through an increase in vessel wall compliance, blood flow can become turbulent and affect such forces as shear and circumferential wall stress. These mechanical perturbations can induce cytoskeletal remodeling in vascular smooth muscle cells resulting in such phenotypes as atherosclerotic plaque destabilization and rupture of the vessel. MMP activity can be controlled at the mRNA/protein levels, tissue inhibitor of metalloproteinases (TIMPs) levels, or through pharmacological intervention [3]. It has been shown that doxycycline, an antibiotic and broad spectrum MMP inhibitor, can decrease the size and incidence of AAAs in both the elastase and angiotensin II-infusion mouse models [2,4]. However, doxycycline did not inhibit established AAA progression in angiotensin II-infused mice [5,6]. It has been shown that doxycycline can increase focal adhesion contact area and that paxillin was concentrated at the cellular edge of rat carotid smooth muscle cells [7]. To this point it is still unclear how MMPs control adhesion dynamics and if these effects are cell specific.

MMP activation can also help to activate the immune response and can be responsible for modulating chemokines such as monocyte chemoattractant protein-3 (MCP-3) in virally-induced myocarditis [8]. Westermann et al. were able to show that MMP-2 knockout mice had elevated myocardial apoptosis, inflammation, and increased mortality compared to wildtype controls [8]. These data indicate that MMP-2 is beneficial in cardiac remodeling. It has also been shown that MMP-2 can degrade myosin light chains during ischemia-reperfusion in the heart indicating that MMPs may also affect contractile proteins [9]. It has been documented that smooth muscle cells, Rous-sarcoma transformed cells, endothelial cells, and macrophages contain podosomes when given certain agonists, such as phorbol esters (PDBu), transforming growth factor-beta (TGF-β), or other possible cytokines [10–14]. Podosomes are actin-rich cores surrounded by myosin and contain a number of actin-binding proteins [13,15–17].

In a study by Varon et al. endothelial cells contain a rosette structure of podosomes when given TGF-β where MMP-9 and -14 are localized to the podosome [14]. The group also noted that when using the synthetic inhibitor, Galardin (also known as GM-6001), that the extracellular matrix (ECM) degradation was abolished and yet podosomes were still able to be formed. In a study by Burgstaller and Gimona, the authors note that A7r5 cells, an embryonic thoracic aorta-derived cell line, also has the ability to degrade the extracellular matrix (ECM) via podosomes and that podosomes may be structures more reminiscent of invadopodia found in virally-induced cancer cell lines [18].

In a study by Xiao et al. human bronchial epithelial cells were shown to contain matrix metalloproteinase-14 (MMP-14, also known as MT1-MMP), MMP-9, and MMP-2. This study showed that MMPs form at the podosome and could degrade extracellular matrix [19]. Furthermore, this group was able to show that protein kinase C signaling was responsible for release and activation of MMP-9 at the podosome [20]. Interestingly, Xiao et al. have shown that podosome formation and proteolytic activity can be uncoupled [20,21].

Another study has found that MMP-2 is co-localized to the podosome in PDBu-stimulated A7r5 smooth muscle cells, however it is not known if MMP inhibition will inhibit podosome formation [22]. Currently, there are 28 identified matrix metalloproteinases and some are only expressed in certain cell types [3]. MMP-2, -9, and -14 are three different MMPs found in vascular smooth muscle [23]. Therefore, the goals of these experiments were to identify specific MMPs in the podosome of the A7r5 cell line and to see if MMP activity was separate from podosome formation. We also wanted to determine the interaction of MMPs with actin stress filaments and microtubules in the A7r5 cell. Furthermore, A7r5 cells were cultured on coverslips and within a Type-I collagen matrix to evaluate differences or similarities in MMP localization in the A7r5 cell.

## Materials & Methods

### Chemicals

Unless otherwise indicated, all chemicals and antibodies were purchased from Sigma (St. Louis, MO).

### Cell culture

A7r5 cells are derived from embryonic rat thoracic aorta and were purchased from ATCC (Manassas, VA). Cells were plated on 75 cm^2^ flasks and grown to approximately 85% confluence at 37 °C in a humidified atmosphere of 5% CO_2_ in air. The cells were maintained in Dulbecco’s modified Eagles medium (DMEM) (Invitrogen, Carlsbad, CA) supplemented with 10% fetal bovine serum, 100 U/mL penicillin G, and 100 μg/mL streptomycin. Media was changed every other day and cells were passaged at least once a week. Passaging was accomplished by addition of a trypsin/EDTA solution (Invitrogen, Carlsbad, CA) in phosphate-buffered saline (PBS) and collection of cells by centrifugation. All experiments were done in triplicate and representative images were taken for each treatment (≥ 30 cells imaged per treatment). Colchicine treatment was given 30 minutes prior to phorbol 12, 13-dibutyrate (PDBu) (10^−5^ M) at a concentration of 40 μg/mL (0.1 μM) (dissolved in water). Latrunculin B was given at 3 μM (dissolved in water) for 30 minutes prior to stimulation with PDBu.

### Fibronectin coating of coverslips

A7r5 cells were plated on unfixed, thinly-layered rhodamine-fibronectin (Cytoskeleton Inc., Denver, CO) coated coverslips at 100 μg/mL for a period of 30 minutes prior to study for MMP-2 and MMP-9 localization.

### Oxyblot method and DHE staining

Samples were prepared for vehicle, 10^−7^, 10^−6^, and 10^−5^ M (PDBu)-stimulated A7r5 cells and lysates were made. The protocol was performed using the Oxyblot Protein Oxidation Detection Kit (Chemicon International, Temecula CA). Densitometry of bands were determined using ImageJ 1.38 program.

Dihydroethidium (DHE) staining was performed on unstimulated and PDBu-stimulated (10^−5^ M) A7r5 cells for 30 minutes at 37 °C and washed with PBS before taken to fluorescent microscope for imaging. Sum pixel intensity divided by cell area was used for calculation of oxidative stress. A total of > 100 cells were evaluated for each treatment.

### Galardin (GM-6001) treatments

A7r5 cells were plated on 22 × 22 mm coverslips (uncoated) (Fischer, Chicago, IL) in 6-well plates (Costar, Corning, NY). PDBu was given before (10^−5^ M) or after Galardin treatment (50 nM–1000 nM) for a period of 30 minutes. Galardin treatment was 30 minutes as well. After treatment, cells were fixed with 2% paraformaldehyde solution and rinsed with PBS. Cells were then permeabilized with 0.1% Triton-X 100 for a period of 20 minutes and then blocked with 5% nonfat milk for 1 hour at 4 °C. Cells were rinsed three times with PBS/0.5% Tween-20 and then incubated with a non-immune Alexa 488 rabbit IgG (negative control, Supplemental Figure 1) or rabbit anti-MMP-2, anti-MMP-9, or anti-MMP-14 (antibody targeted to hinge region) overnight at 4 °C. Cells were rinsed three times and an anti-rabbit Alexa 488 IgG or Alexa 568 IgG was added for 1 hour at 4 °C. Cells were rinsed and an anti-mouse α-actin antibody (clone 1A4) was given for 1 hour followed by an Alexa 488, 568 or 647 IgG secondary. A TRITC-labeled anti-mouse paxillin antibody (BD Biosciences, clone 349/Paxillin) was also evaluated with MMP-2 staining. Cell counts of podosome development were recorded by two independent observers who were blinded to the treatment groups and a total of 300 or more cells were evaluated. Proteolytic activity was also measured by use of gelatin-FITC using the method of Chen et al. and Bowden et al. [24,25]. A7r5 cells were pre-treated with either 0, 250, 500, or 1000 nM of Galardin for 30 minutes and then given PDBu (10^−5^ M) for 30 minutes. Degradation spots were counted via ImageJ plugin (cell counter).

### Western blots and Gel zymography

A7r5 cells were plated on 250 cm^2^ plates and allowed to grow to 85% confluence. Cells were treated and then rinsed in warm PBS before extracting protein with ice-cold buffer, pH 7.5 (50 mM Tris-HCl, 1M NaCl, 2M urea, 0.1% Brij-35) with protease inhibitor cocktail (Roche, Palo Alto, CA). Protein extracts were sonicated for 10 seconds on a low setting and spun at 14,000 g’s to remove particulate. Protein determination was performed with BCA kits in 96-well plates (Pierce, Rockford, IL). Equal amounts of protein with dithiothreitol (DTT) were ran on 10% SDS-PAGE gels and blotted with the same antibodies used in immunofluorescent studies. GAPDH (Ambion, Austin, TX) antibody was used to evaluate protein loading. Gel zymography was performed according to Zhang HJ et al. with protein extracts without DTT and ran on 10% SDS-PAGE gels supplemented with 1.5 mg/mL α-casein [26]. Gels were run at 80V for a period of 2.5 hours. After electrophoresis, gels were washed twice with 2.5% Triton-X 100, 50 mM Tris-HCl, pH 8.0 at 37 °C for 30 minutes. Gels were transferred to substrate buffer (50 mM Tris-HCl, 10 mM CaCl_2_, 0.02% sodium azide, pH 8.5) for 24 hours at 37 °C. Gels were stained with 0.5% CooMassie blue R-250 and destained with 10% methanol/10% acetic acid until clear bands could be detected against the blue background.

### RT-PCR

RNA extraction was performed using TriZol reagent and the 280/260 nm ratio was used to determine RNA concentration by spec-trophotometry. One-step RT-PCR was performed with the Platinum RT kit from Invitrogen (Carlsbad, CA). We used equal amounts of total RNA from each sample (100 nanograms of RNA). The thermocycler protocol was as follows: 45 °C for 30 min, 94 °C for 2 min, then 40 cycles of 94 °C for 1 min, 60 °C (for MMP-2) or 56 °C (for MMP-9 & 14) for 1 min, and 72 °C for 1 min, followed by 72 °C for 10 min, then 4 °C until we removed the samples. Primer sequence for MMP-14 and MMP-2 were purchased from BIOMOL (Plymouth, PA) and were as followed: (MMP-14, forward 5′- GTGCCCTATGCCTACATCCG, reverse 5′-CAGCCACCAAGAAGATGTCA, MMP-2, forward 5′-CCCCTATCTACACCTACACCAAGAAC, reverse 5′-CATTC-CAGGAGTCTGCGATGAGC). MMP-9 primers were synthesized by MWG Biotech (Huntsville, AL) and were: (forward, 5′- TCGTG-GCTCTAAACCTGACC, reverse, 5′-TTCCTCCGTGATTCGAGAAC). Gel loading buffer was added to each sample and aliquots were analyzed with gels composed of 1.5% Agarose in 1X TAE. Gels were ran for approximately 45 min at 100 V and then stained in an ETBr solution and examined with a UV light. Densitometric values were determined using ImageJ 1.38 program.

### Type I collagen matrix gels

A7r5 cells were mixed in a collagen gel using the protocol described in Li et al. with some modifications [27]. The gels consisted of 52% 3 mg/mL collagen type I (Inamed, Fremont, CA), 9.5% 0.1 M NaOH, 9.5% of 10X DMEM with 200 mM HEPES and 2.2% NaHCO_3_ (Invitrogen, Carlsbad, CA), 9.5% FBS, and 19.5% complete medium with a cell density of 1× 10^6^ cells/mL. Twelve-well plates were used and 0.5 mLs of collagen gel was added to each well. Each well was treated with 5% BSA before addition of collagen gel to allow detachment of the gel upon contraction. In some cases, a bent spatula was used to lift the gel off of culture dishes. Gels were allowed to contract and remodel for 48 hours and then gels were treated with PDBu, Galardin, or a combination of both for an additional 24 hours prior to scanning the plates using a Epson 2580 photo scanner to determine the area of contraction both before and after stimulation.

## Statistics

Data was analyzed using Sigma Stat 3.1 software and t-tests were utilized to determine significant differences at p < 0.05 level. Oneway ANOVA was used to determine differences among the groups in Figures 7 and 8 and reported at p < 0.05.

## Results

### Activation of matrix metalloproteinases-14, -9, and -2 occur at the protein level under phorbol dibutyrate (PDBu) stimulation

**Figure 1:**
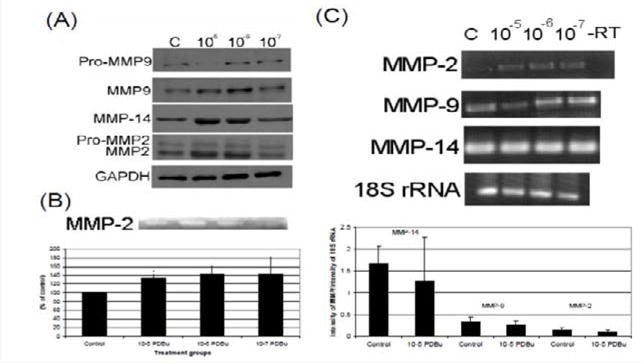
**(A)** Western blots, **(B)** Gel zymography and **(C)** RT-PCR. Analysis of A7r5 cells under various concentrations of phorbol esters (10^−7^–10^−5^ M). Data suggests that increase in the active forms of MMP-2, -9, and -14 are a result of a post-translational modification and not an increase in mRNA abundance. A Student’s t-test was used to compare control versus PDBu-stimulation (10^5^ M). Asterisks in **(B)** indicates significance at p < 0.05. Data is represented as means ± SEM.

In order to understand MMP activation in the A7r5 cell, different concentrations of PDBu were utilized and Western blotting of MMPs and gel zymography of MMP-2 activity were performed (Figure 1A and 1B). Active forms of MMPs were present with all concentrations of PDBu, however it was significantly higher with PDBu given at 10^−5^ M after 30 minutes of stimulation. Messenger RNA (mRNA) expression was also performed and PDBu at 10^−5^ M did not change mRNA expression of MMPs in A7r5 cells (Figure 1C). Therefore, PDBu was given at 10^−5^ M for 30 minutes for all subsequent imaging studies.

Specificity of the MMP antibodies were done by incubating a non-immune rabbit IgG Alexa 488 antibody on A7r5 cells and imaged with the same exposure settings as MMP localization studies. A small amount of non-specific nuclear signal was detected however no such detection was seen at the podosome (Supplementary Figure 1).

### PDBu stimulation induces co-localization of MMP-14, -9, and -12 with α-actin not β-tubulin in A7r5 smooth muscle cells

**Figure 2:**
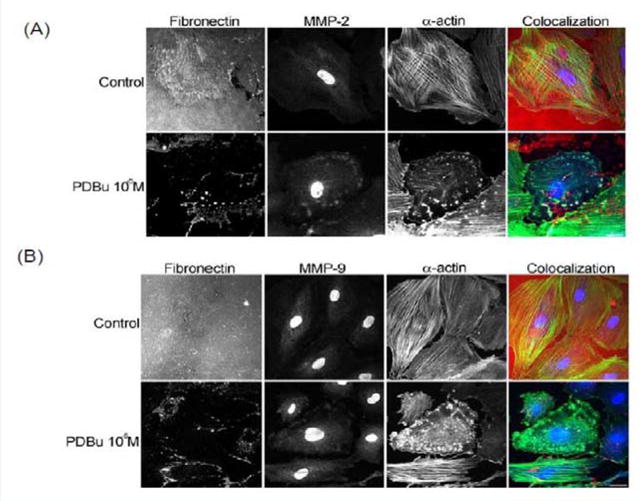
**(A)** Colocalization images of MMP-2 and **(B)** Colocalization images of MMP-9. Under unstimulated and PDBu-stimulated conditions (10^−5^ M) in the A7r5 cell. Cell study was performed in triplicate and at least 30 cells were observed to have this phenotype. Rhodamine-conjugated fibronectin (100 μg) was used to thinly coat coverslips and cells were contracted with PDBu for a period of 30 minutes at 37 °C. Blue represents MMP-2/9 staining, red represents fibronectin staining, and green represents α-actin staining. Scale bar represents 20 μm.

In order to evaluate MMP localization, coverslips were thinly coated with rhodamine conjugated fibronectin. In unstimulated conditions, A7r5 cells degraded only a small portion of the fibronectin, however in the presence of PDBu (10^−5^ M), substantial degradation of the fibronectin was evident (Figure 2). MMP-2, -9, and -14 were found to be localized with α-actin in the podosome of the A7r5 cell (Figure 2) (Supplemental Figure 2).

**Figure 3:**
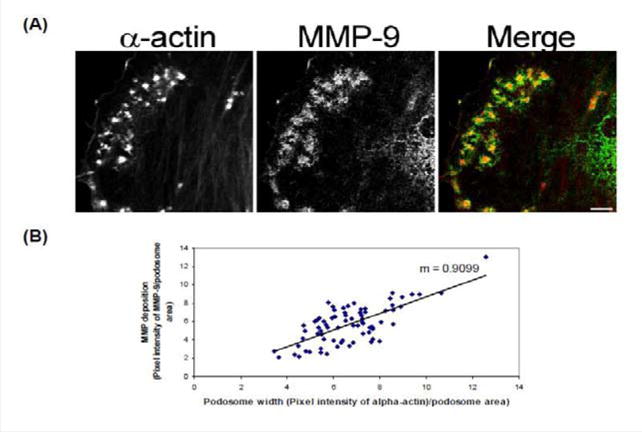
**(A)** High-power magnification (1000×) of podosomes immunostained for MMP-9 and α-actin in the A7r5 cell. Ten different cell sections and 73 individual podsomes were evaluated. **(B)** Linear slope analysis of pixel intensity of α-actin within the podosome (X-axis) with pixel intensity of MMP-9 within the podosome (Y-axis). Slope of the line was 0.9. Green represents MMP-9 staining and red represents α-actin staining. Scale bar represents 5 μm.

**Figure 4:**
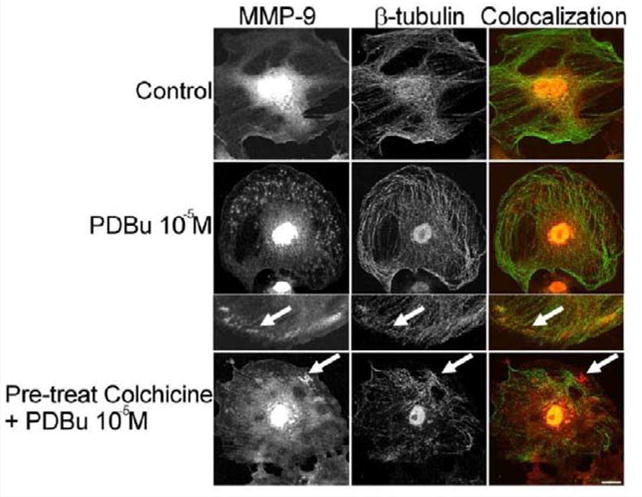
Colocalization images of MMP-9 and β-tubulin under unstimulated (control), PDBu (10^−5^ M) and pre-treatment with a microtubule-depolymerization agent (colchicine at 0.1 μM) in the A7r5 cell. Cell study was performed in triplicate and at least 30 cells were observed to have this phenotype. White arrows in upper panels represent that MMP-9 does not localize to β-tubulin in the podosome. White arrows in bottom panels indicate that MMP-9 still localizes to a cluster of podosomes despite the absence of microtubules. Green represents β- tubulin staining and red represents MMP-9 staining. Scale bar represents 20 μm.

In order to determine how MMPs were translocating to the podosome, a series of experiments were performed. First, high magnification images of podosomes (PDBu 10^−5^ M) were taken and pixel intensities of α-actin were compared to pixel intensities of MMP-9 (Figure 3A). From 10 different cell sections and 73 individual podosomes, a best-fit line was created and a slope of 0.9 was obtained. This data suggests that as the pixel intensity of α-actin increased so did MMP-9 deposition (Figure 3B). A similar analysis was also performed for MMP-2 and paxillin. The interaction between MMP-2 and paxillin was less in comparison to α-actin since the slope of the line had a greater deviation from 1 (m = 1.6), therefore suggesting that the α-actin/MMP interaction is closer than the paxillin/MMP interaction (Supplementary Figure 4). Beta(β)-tubulin is a main component of microtubules and is responsible for the translocation of PKCα to the cell membrane [28,29]. PKCα is a signaling molecule involved in the formation of podosomes [20,28,29]. Dual staining of β-tubulin with MMP-9 indicated no colocalization between the two proteins (Figure 4). Thirty minutes of pre-treatment with colchicine has been shown to sufficiently cause the breakdown of the microtubular system (Figure 4) [28]. However, at high concentrations of PDBu (10^−5^ M), A7r5 cells are able to sufficiently remodel and form podosomes (Supplementary Figure 5). In Figure 4, the bottom set of white arrows indicate podosome localization of MMP-9 despite breakdown of the microtubules, suggesting that microtubules are not needed for MMP-9 translocation. Alpha-actin filaments were also evaluated under colchicine treatment and found to not change in structure or podosome formation (Supplementary Figure 5).

### Actin depolymerization blocks podosome formation and prevents interaction between MMP-9 and α-actin

**Figure 5:**
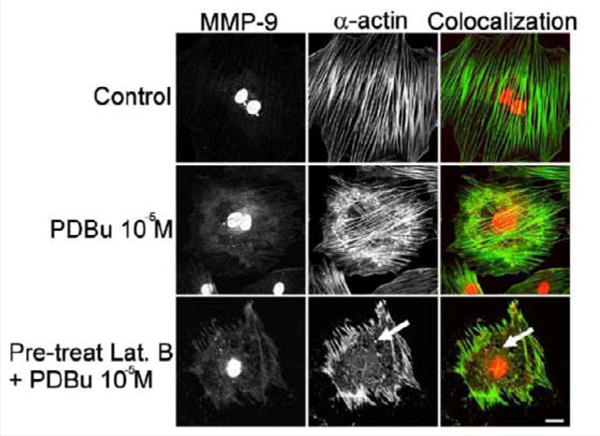
Colocalization images of MMP-9 and α-actin in the A7r5 cell under unstimulated (control), PDBu (10^−5^ M) and Latrunculin B pre-treatment (3 μM). Cell study was performed in triplicate and at least 30 cells were observed to have this phenotype. Bottom panel shows that in the presence of an actindepolymerization agent MMP-9 shows a dispersed arrangement in the absence of α-actin filaments (white arrows). Green represents α-actin staining and red represents MMP-9 staining. Scale bar represents 20 μm.

Latrunculin B is an actin depolymerization agent that binds to G-actin monomers and dimers preventing actin polymerization [30]. Actin polymerization is also a necessary step in the formation of podosomes [31]. Pre-treatment of Latrunculin B (3 μM) caused sufficient dissolution of the actin filaments and prevented podosome formation (Figure 5). Confocal images also showed dispersion of MMP-9 in the absence of the actin filaments (white arrows, Figure 5). This data seems to suggest that MMP-9 is tethered to the α-actin filament.

### Tissue inhibitors of matrix metalloproteinases (TIMPs) are localized to podosomes and interact with α-actin in A7r5 smooth muscle cells

**Figure 6:**
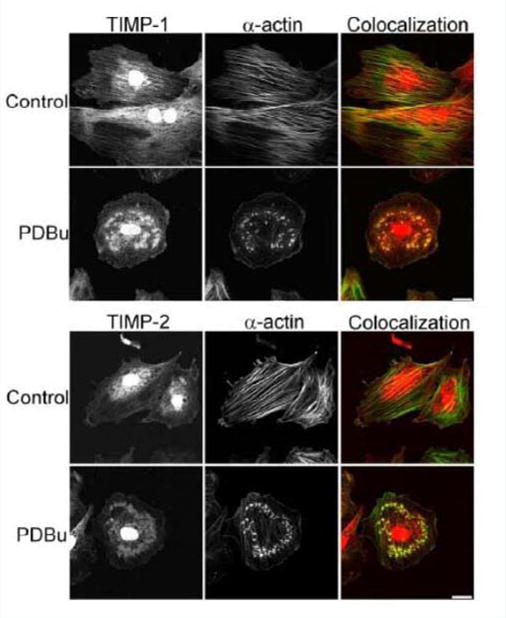
TIMP-1 and -2 colocalization with α-actin under control (unstimulated) and PDBu-stimulated (10^−5^ M) conditions in the A7r5 cell. Cell study was performed in triplicate and at least 30 cells were observed to have this phenotype. Both TIMP-1 and -2 localize to the podosome of the A7r5 cell. Green represents α-actin staining and red represents TIMP-1/2 staining. Scale bar represents 20 μm.

Tissue inhibitors of matrix metalloproteinases (TIMPs) are known to bind to pro and active forms of MMPs to prevent inappropriate degradation [3]. MMP-9 preferentially binds to TIMP-1, while MMP-14 and -2 bind to TIMP-2 [3]. Colocalization studies indicate interaction between α-actin filaments and TIMP-1 and -2 (Figure 6). TIMP-1/2 were also localized to the podosome (Figure 6).

### MMP inhibition uncouples matrix degradation from podosome formation in the A7r5 smooth muscle cell

**Figure 7:**
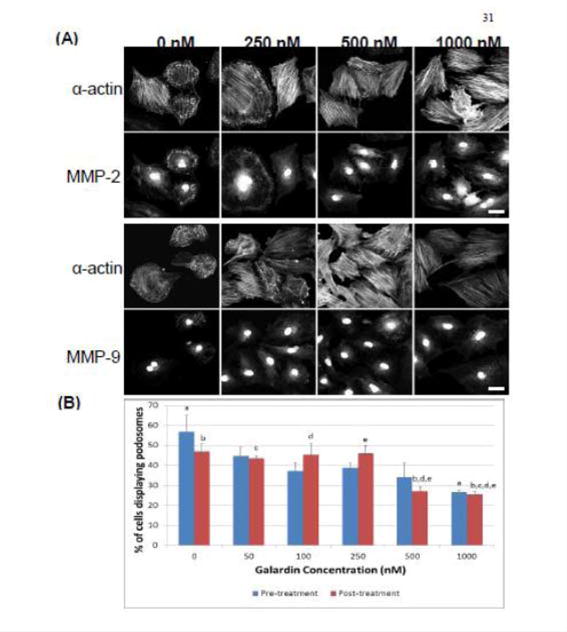
**(A)** Confocal images of MMP-2 and MMP-9 with pre-treatment of a broad-spectrum MMP inhibitor, Galardin, under PDBu-stimulated conditions (10^−5^ M). **(B)** Cell counts of A7r5 cells displaying podosomes with various concentrations of Galardin under pre-treatment and post-treatment conditions of PDBu-stimulation. Cell study was performed in triplicate and at least 300 cells were evaluated by two different observers. A one-way ANOVA was utilized in analysis of data. Identical letters indicate significance between groups at p < 0.05. Data is represented as means ± SEM.

**Figure 8:**
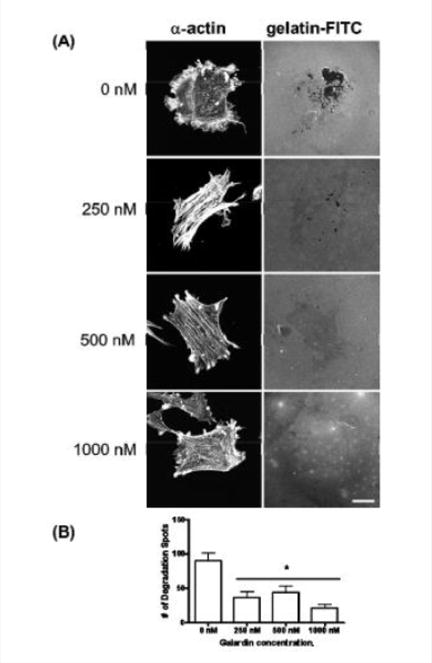
**(A)** A7r5 cells grown on gelatin-FITC under pre-treatment of a broad spectrum MMP inhibitor, Galardin, under PDBu-stimulated conditions (10^−5^ M). Cell study was performed in triplicate and at least 30 cells were observed to have this phenotype. Note that gelatin degradation decreased under all concentrations of Galardin. This indicates that matrix degradation can be uncoupled from podosome formation. In **(B)**, matrix degradation spots were counted and quantified under increasing concentrations of Galardin. A one-way ANOVA was utilized in analysis of data. Asterisk in **(B)** indicates significance at p < 0.05. Data is represented as means ± SEM. Scale bar represents 20 μm.

Galardin, also known as Ilomastat and GM-6001, is a broad MMP inhibitor and was used to evaluate if MMPs were important for podosome initiation and function (Figures 7 and 8). Thirty minutes of pre-treatment with Galardin, showed a dose-dependent decrease in cells displaying podosomes (Figure 7B) and cells stained with MMP-2 or MMP-9 (Figure 7A) did not show translocation to the podosome in regards to the highest concentrations of Galardin given. Post-treatment of Galardin (Figure 7B) also showed decreases of cells displaying podosomes at 500 nM and 1000 nM, indicating that MMPs are needed in the initiation and functioning of podosomes in the A7r5 cell. A7r5 cells were also evaluated on gelatin-FITC to evaluate proteolytic degradation of matrix. Cells pre-treated with all doses of Galardin showed significant decreases in degradation indicating that Galardin was effective in blocking matrix degradation (Figure 8A and 8B). This data indicates that proteolytic degradation of matrix can be uncoupled from podosome formation.

### MMP inhibition decreases collagen matrix gel contraction and influences cell spreading and α-actin filament organization in the A7r5 smooth muscle cell

**Figure 9:**
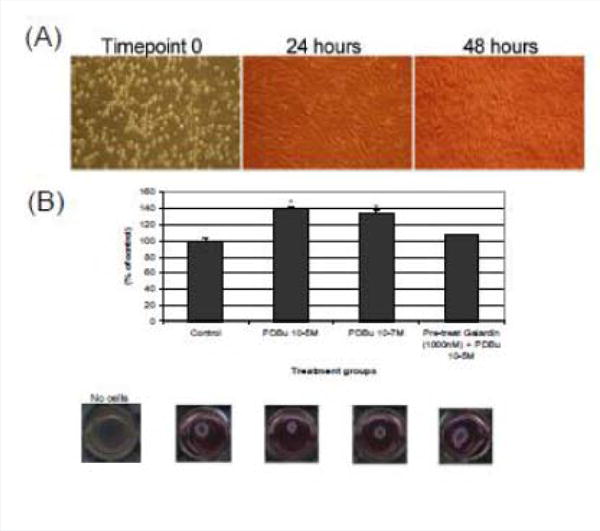
**(A)** Phase-contrast images (100× magnification) of A7r5 cells seeded in a Type-I collagen matrix. Cells were allowed to remodel the collagen for 48 hours prior to experimentation. In **(B)**, cells were then left unstimulated (control), treated with PDBu (10^−7^ M or 10^−5^ M) or pre-treated with Galardin (1000 nM) for 30 minutes and then given phorbol (10^−5^ M) for an additional 24 hours. The area of the collagen was calculated before and after stimulation and based as a percent of control. Cell study was performed in triplicate. Asterisks represent a significant difference at p < 0.001. Data is represented as means ± SEM.

**Figure 10:**
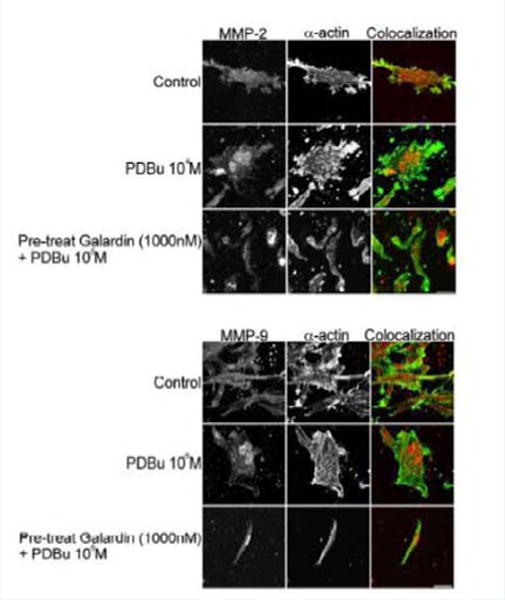
Colocalization of MMP-2 or MMP-9 with α-actin in A7r5 cells within a Type-I collagen matrix. Cell study was performed in triplicate and at least 30 cells were observed to have this phenotype. Note that cells pre-treated with Galardin (1000 nM) showed less cell spreading and focal adhesions in comparison to control and PDBu-stimulated (10^−5^ M) A7r5 cells. Green represents α-actin staining and red represents MMP-2/9 staining. Scale bar represents 20 μm.

A7r5 cells were grown in a Type I collagen matrix to evaluate the development of gel contraction in the presence of PDBu (10^−5^ M, 10^−7^ M) and pre-treatment of Galardin (1000 nM) during a 24-hour stimulation. It has been shown that under the presence of serum, smooth muscle cells will remodel the collagen matrix and cause it to contract (Figure 9B). We allowed cells to remodel the matrix for 48 hours (Figure 9A) and then gave PDBu (10^−5^ M, 10^−7^ M) or pre-treatment of Galardin (1000 nM) for an additional 24 hours. Cells given phorbol esters contracted an additional 40% in comparison to controls and pre-treatment with Galardin caused only a 7% development in gel contraction. These results suggest that MMPs play an important role in 3-D gel contraction and additional cell staining was conducted on the gel matrix in order to evaluate MMP-9 and -2 localization with α-actin (Figure 10). Both MMP-9 and -2 were found to colocalize with α-actin and Galardin influenced cell spreading and remodeling within the collagen matrix. These data indicate that MMPs are necessary for remodeling the collagen matrix and help actin filament organization in the A7r5 smooth muscle cell.

### PDBu does not significantly increase oxidative stress in the A7r5 smooth muscle cell

A7r5 cells were also stimulated for 30 minutes with various concentrations of PDBu and an Oxyblot was performed to detect protein carbonyls accumulation (Supplemental Figure 3A and 3B). A7r5 cells were also treated with DHE under unstimulated and PDBu-stimulated conditions for detection of superoxide radicals (Supplemental Figure 3C). The data indicates that all concentrations of PDBu induce a higher level of oxidative stress than unstimulated A7r5 cells, however this data was not significant. This data suggests that PDBu does not significantly increase oxidative stress in the A7r5 cell. These studies indicate that MMP-14, -9, and -2 are localized to the α-actin-rich podosome and that microtubules are not involved in this process. Using a broad spectrum MMP inhibitor, Galardin blocked matrix degradation, prevented MMP-9 and -2 localization to the podosome, and decreased matrix remodeling in a Type I collagen gel.

## Discussion

MMPs are known to have pathological effects in cancer metastasis, atherosclerosis, and AAA development [1]. In regards to vascular smooth muscle, MMP-14, -9, and -2 have been found and various functions have been described for each MMP [3]. For example, MMP-14 was found to be involved in vascular smooth muscle cell migration and collagenase activity in media explants of mice [23]. MMP-14 could then transmit signals from the extracellular environment and activate intracellular MMPs, such as MMP-9 and MMP-2. Western blot analysis in A7r5 cells revealed that PDBu produced the active forms of all MMPs with the highest at 10^−5^ M. Gel zymography also showed a significant increase in MMP-2 activity at this highest concentration of PDBu. There were not any increases in mRNA concentrations indicating that this type of MMP activation was post-transcriptional. Similar studies have shown differences in actin remodeling under lower PDBu concentrations [11]. Fultz et al. has shown that α-actin stress fibers remodel into podosomes whereas the β-actin filaments do not [11]. Since we wanted to understand the localization and interaction of MMPs to podosomes, a high concentration of PDBu was used to study the role of both actin isoforms with MMP localization. Another study has also used the same concentration of PDBu to study podosome dynamics [32]. This type of signaling could cause excessive degradation of the extracellular substrate as seen with the A7r5 cells on fibronectin thinly coated coverslips (Figure 2) (Supplemental Figure 2). Since podosomes contain these MMPs, it is now thought that podosomes may be degradation-type structures [33,34]. Xiao et al. has shown that knockdown of MMP-14 and -9, but not MMP-2, can prevent matrix degradation of PDBu-induced podosomes in BEAS2B cells [20]. Even though matrix degradation can be blocked, podosome formation can still occur [14,19]. Our study also indicates that podosomes can form even though matrix degradation is blocked (Figures 7 and 8). MMPs also have differences in interaction when compared to different proteins. Interaction between α-actin and MMP-9 were clearly stronger than interaction between paxillin and MMP-2 (Figure 2) (Supplementary Figure 4).

Our new finding is that MMPs look to be tethered to the actin cytoskeleton and not microtubules in the A7r5 cell (Figures 2–4). Latrunculin B binds to actin monomers preventing polymerization of actin filaments. Pre-treatment of latrunculin B prevented MMP-9 localization to the podosome. Other studies have also shown that glioma cells treated with cytochalasin D, another actin depolymerization agent, can influence MMP-9 expression and cancer cell invasion [35]. These effects were independent of microtubules as well, since no effect of MMP-9 expression was evident under colchicine treatment [35]. New evidence has shown that actin polymerization can be controlled by the c-Abl-cortactin pathway [36]. c-Abl is a proto-oncogene that functions as a non-receptor tyrosine kinase that can control smooth muscle contraction and migration. Future studies could be designed to determine if the c-Abl-cortactin pathway plays a role in MMPs activation and if inhibitors of MMPs influence this pathway. Other studies, however, have shown that colchicine treatment can influence MMP-2 activity and MMP-14 gene expression in osteosarcoma cells [37]. These studies indicate that microtubules are not involved since there was no colocalization between the two proteins and that colchicine did not disrupt MMP localization to the podosome. Furthermore, colchicine treatment does not influence actin filament structure or prevent podosome development at this inparticular PDBu concentration.

Podosome formation also occurs through PKCα activation and it has now been shown that PKC activation can influence MMP release and activation [20]. MMP activation does not seem to occur through an increase in transcription, but possibly by a post-translational modification and this has been shown in a previous study to occur in epithelial cells [20]. TIMP-1/2 were found to be localized to the podosome, however it is unclear how this signaling is regulated under PDBu-stimulated conditions in the A7r5 cell. Future studies could be to look at the interaction of TIMPs with MMPs in the podosome with the use of fluorescently-tagged proteins. Recent data also indicates that Src kinase can induce podosomes through tyrosine phosphorylation of G-protein coupled receptor kinase 2-interacting protein 1 (GIT1) and this can activate phospholipase C-gamma (γ) [38]. It will be of interest to see how other signaling pathways might modulate MMP expression and/or activity.

Galardin, also known as GM–6001, is a broad-spectrum MMP inhibitor and found to prevent degradation of the extracellular matrix [14]. Varon et al. showed that podosomes in endothelial cells still form in the presence of Galardin [14]. Xiao et al. have also shown that proteolytic activity can be uncoupled from podosome formation in bronchial epithelial cells [19–21]. Our study also confirms these findings in the A7r5 smooth muscle cell. Pre- and post-treatment with Galardin showed that podosome formation and podosome functioning (Figure 7B) decreased the percentage of A7r5 cells displaying podosomes. However, lower concentrations of Galardin were able to decrease matrix degradation despite podosome formation (Figure 8B). Future studies should address the role of A7r5 cell migration under treatment with Galardin as many studies have shown that smooth muscle migration is important in the development of cardiovascular disease [36,39,40]. These results suggest that MMPs play an important role in smooth muscle cell migration and contraction.

Smooth muscle cells have also been cultured in 3-D collagen matrices [27]. This mimics the environment present in a blood vessel and allows the study of smooth muscle cell spreading and focal adhesion dynamics that could be different than cells grown *in vitro*. Here we show that A7r5 cells spread and form adhesive attachments to the collagen under the presence of serum and phorbol stimulation, but this cell spreading/adhesion is absent in Galardin-treated collagen gels (Figures 9 and 10). Furthermore, Galardin treatment prevents the ability for the collagen gel to contract (Figure 9B). It has been shown that deoxycycline can also inhibit collagen cell contraction of rat carotid smooth muscle cells [7]. Defawe et al. has shown that MMP-9 can induce a bi-phasic response in smooth muscle cells where low concentrations of exogenous MMP-9 enhanced gel contraction but high concentrations inhibited gel contraction [41]. Our study would seem to indicate that MMP inhibition would inhibit gel remodeling, however this could be due to the Galardin concentration used.

These studies provide evidence that MMPs are important for podosomes and that this is an α-actin-dependent process. Furthermore, MMPs are important for podosome development and focal adhesion dynamics in the A7r5 cell.

## Supplementary Material

JCB-2332-3671-05-0020

## References

[1] Galis ZS, Khatri JJ (2002). Matrix metalloproteinases in vascular remodeling and atherogenesis: the good, the bad, and the ugly. Circ Res.

[2] Manning MW, Cassis LA, Daugherty A (2003). Differential effects of doxycycline, a broad-spectrum matrix metalloproteinase inhibitor, on angiotensin II-induced atherosclerosis and abdominal aortic aneurysms. Arterioscler Thromb Vasc Biol.

[3] Woessner JF (2002). MMPs and TIMPs–an historical perspective. Mol Biotechnol.

[4] Bartoli MA, Parodi FE, Chu J, Pagano MB, Mao D (2006). Localized administration of doxycycline suppresses aortic dilatation in an experimental mouse model of abdominal aortic aneurysm. Ann Vasc Surg.

[5] Iida Y, Xu B, Schultz GM, Chow V, White JJ (2012). Efficacy and mechanism of angiotensin II receptor blocker treatment in experimental abdominal aortic aneurysms. PLoS One.

[6] Xie X, Lu H, Moorleghen JJ, Howatt DA, Rateri DL (2012). Doxycycline does not influence established abdominal aortic aneurysms in angiotensin II-infused mice. PLoS One.

[7] Franco C, Ho B, Mulholland D, Hou G, Islam M (2006). Doxycycline alters vascular smooth muscle cell adhesion, migration, and reorganization of fibrillar collagen matrices. Am J Pathol.

[8] Westermann D, Savvatis K, Lindner D, Zietsch C, Becher PM (2011). Reduced degradation of the chemokine MCP-3 by matrix metalloproteinase-2 exacerbates myocardial inflammation in experimental viral cardiomyopathy. Circulation.

[9] Cadete VJ, Sawicka J, Jaswal JS, Lopaschuk GD, Schulz R (2012). Ischemia/reperfusion-induced myosin light chain 1 phosphorylation increases its degradation by matrix metalloproteinase 2. FEBS J.

[10] Brandt D, Gimona M, Hillmann M, Haller H, Mischak H (2002). Protein kinase C induces actin reorganization via a Src- and Rho-dependent pathway. J Biol Chem.

[11] Fultz ME, Li C, Geng W, Wright GL (2000). Remodeling of the actin cytoskeleton in the contracting A7r5 smooth muscle cell. J Muscle Res Cell Motil.

[12] Gatesman A, Walker VG, Baisden JM, Weed SA, Flynn DC (2004). Protein kinase Calpha activates c-Src and induces podosome formation via AFAP-110. Mol Cell Biol.

[13] Linder S, Kopp P (2005). Podosomes at a glance. J Cell Sci.

[14] Varon C, Tatin F, Moreau V, Van Obberghen-Schilling E, Fernandez-Sauze S (2006). Transforming growth factor beta induces rosettes of podosomes in primary aortic endothelial cells. Mol Cell Biol.

[15] Fultz ME, Wright GL (2003). Myosin remodelling in the contracting A7r5 smooth muscle cell. Acta Physiol Scand.

[16] Gimona M, Buccione R, Courtneidge SA, Linder S (2008). Assembly and biological role of podosomes and invadopodia. Curr Opin Cell Biol.

[17] Thatcher SE, Fultz ME, Tanaka H, Hagiwara H, Zhang HL (2011). Myosin light chain kinase/actin interaction in phorbol dibutyrate-stimulated smooth muscle cells. J Pharmacol Sci.

[18] Burgstaller G, Gimona M (2005). Podosome-mediated matrix resorption and cell motility in vascular smooth muscle cells. Am J Physiol Heart Circ Physiol.

[19] Xiao H, Eves R, Yeh C, Kan W, Xu F (2009). Phorbol ester-induced podosomes in normal human bronchial epithelial cells. J Cell Physiol.

[20] Xiao H, Bai XH, Kapus A, Lu WY, Mak AS (2010). The protein kinase C cascade regulates recruitment of matrix metalloprotease 9 to podosomes and its release and activation. Mol Cell Biol.

[21] Xiao H, Bai XH, Wang Y, Kim H, Mak AS (2013). MEK/ERK pathway mediates PKC activation-induced recruitment of PKCζ and MMP-9 to podosomes. J Cell Physiol.

[22] Gu Z, Kordowska J, Williams GL, Wang CL, Hai CM (2007). Erk1/2 MAPK and caldesmon differentially regulate podosome dynamics in A7r5 vascular smooth muscle cells. Exp Cell Res.

[23] Filippov S, Koenig GC, Chun TH, Hotary KB, Ota I (2005). MT1-matrix metalloproteinase directs arterial wall invasion and neointima formation by vascular smooth muscle cells. J Exp Med.

[24] Bowden ET, Coopman PJ, Mueller SC (2001). Invadopodia: unique methods for measurement of extracellular matrix degradation *in vitro*. Methods Cell Biol.

[25] Chen WT, Yeh Y, Nakahara H (1994). An *in vitro* cell invasion assay: determination of cell surface proteolytic activity that degrades extracellular matrix. J Tissue Cult Methods.

[26] Zhang HJ, Zhao W, Venkataraman S, Robbins ME, Buettner GR (2002). Activation of matrix metalloproteinase-2 by overexpression of manganese superoxide dismutase in human breast cancer MCF-7 cells involves reactive oxygen species. J Biol Chem.

[27] Li S, Moon JJ, Miao H, Jin G, Chen BP (2003). Signal transduction in matrix contraction and the migration of vascular smooth muscle cells in three-dimensional matrix. J Vasc Res.

[28] Dykes AC, Fultz ME, Norton ML, Wright GL (2003). Microtubule-dependent PKC-alpha localization in A7r5 smooth muscle cells. Am J Physiol Cell Physiol.

[29] Li C, Fultz ME, Geng W, Ohno S, Norton M (2001). Concentration-dependent phorbol stimulation of PKCalpha localization at the nucleus or subplasmalemma in A7r5 cells. Pflugers Arch.

[30] Danninger C, Gimona M (2000). Live dynamics of GFP-calponin: isoform-specific modulation of the actin cytoskeleton and autoregulation by C-terminal sequences. J Cell Sci.

[31] Kaverina I, Stradal TE, Gimona M (2003). Podosome formation in cultured A7r5 vascular smooth muscle cells requires Arp2/3-dependent de-novo actin polymerization at discrete microdomains. J Cell Sci.

[32] Dykes AC, Wright GL (2007). Down-regulation of calponin destabilizes actin cytoskeletal structure in A7r5 cells. Can J Physiol Pharmacol.

[33] Lener T, Burgstaller G, Crimaldi L, Lach S, Gimona M (2006). Matrix-degrading podosomes in smooth muscle cells. Eur J Cell Biol.

[34] Linder S (2007). The matrix corroded: podosomes and invadopodia in extracellular matrix degradation. Trends Cell Biol.

[35] Chintala SK, Kyritsis AP, Mohan PM, Mohanam S, Sawaya R (1999). Altered actin cytoskeleton and inhibition of matrix metalloproteinase expression by vanadate and phenylarsine oxide, inhibitors of phosphotyrosine phosphatases: modulation of migration and invasion of human malignant glioma cells. Mol Carcinog.

[36] Tang DD, Gerlach BD (2017). The roles and regulation of the actin cytoskeleton, intermediate filaments and microtubules in smooth muscle cell migration. Respir Res.

[37] Loennechen T, Mathisen B, Hansen J, Lindstad RI, El-Gewely SA (2003). Colchicine induces membrane-associated activation of matrix metalloproteinase-2 in osteosarcoma cells in an S100A4-independent manner. Biochem Pharmacol.

[38] Wang J, Yin G, Menon P, Pang J, Smolock EM (2010). Phosphorylation of G protein-coupled receptor kinase 2-interacting protein 1 tyrosine 392 is required for phospholipase C-gamma activation and podosome formation in vascular smooth muscle cells. Arterioscler Thromb Vasc Biol.

[39] Gerthoffer WT (2007). Mechanisms of vascular smooth muscle cell migration. Circ Res.

[40] Cleary RA, Wang R, Waqar O, Singer HA, Tang DD (2014). Role of c-Abl tyrosine kinase in smooth muscle cell migration. Am J Physiol Cell Physiol.

[41] Defawe OD, Kenagy RD, Choi C, Wan SY, Deroanne C (2005). MMP-9 regulates both positively and negatively collagen gel contraction: a nonproteolytic function of MMP-9. Cardiovasc Res.

