# Supplementary material for: Matrix Metalloproteinases -14, -9 and -2 are Localized to the Podosome and Involved in Podosome Development in the A7r5 Smooth Muscle Cell
Source: J Cardiobiol. Author manuscript; Available in PMC 2019 Mar 27. (PMC6436839; doi:10.13188/2332-3671.1000020)

Supplementary Information

Supplemental Figure 1: Non-immune Alexa 488 rabbit IgG incubated overnight at 4 °C to evaluate specificity of rabbit antibodies. Cells were contracted with PDBu (10^{-5} M) to determine non-specific staining at the podosome. Note that a little perinuclear staining could be detected using the same settings for analysis of MMPs. Nuclei were stained with DAPI. Scale bar represents 20 µm.

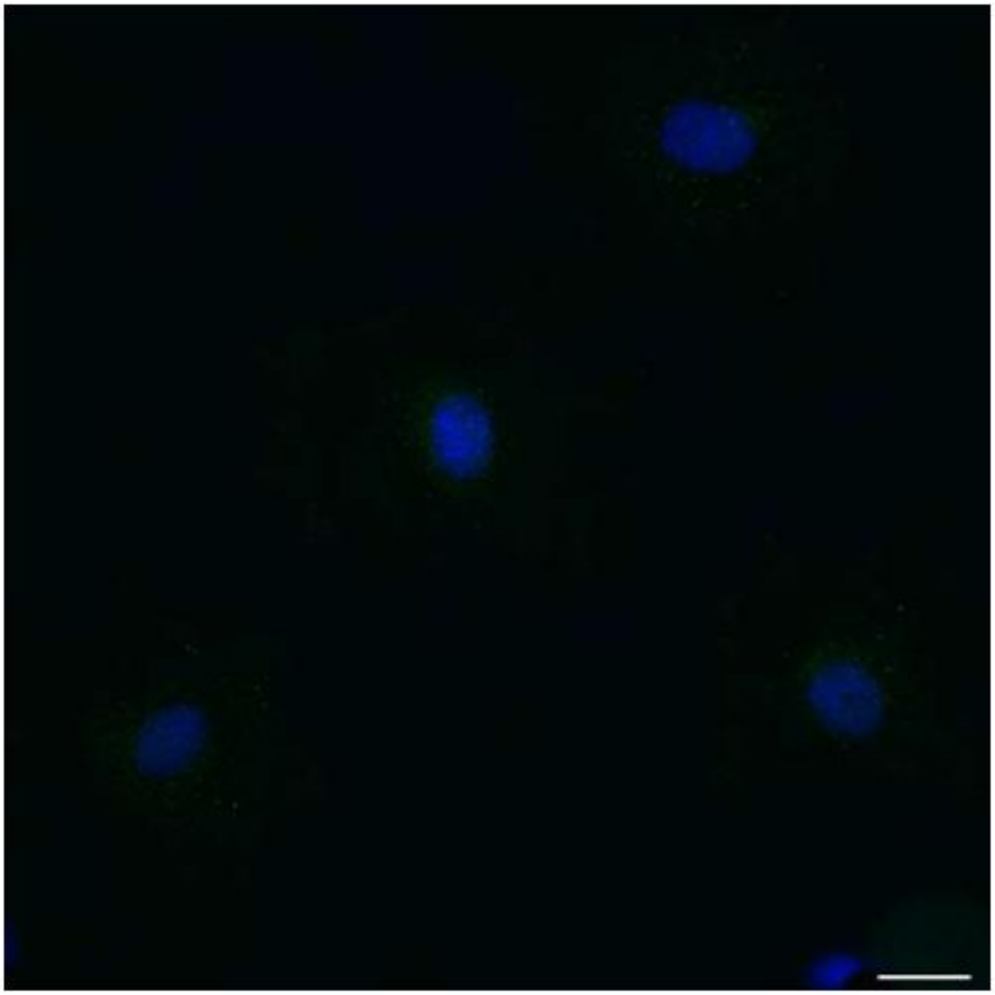

Supplemental Figure 2: MMP-14 and α-actin colocalization in A7r5 cells on a thinly-coated rhodamine-conjugated fibronectin substrate. Note that MMP-14 behaves similarly to MMP-2 and -9 in the A7r5 smooth muscle cell. Cell study was performed in triplicate and at least 30 cells were observed for this phenotype. Red represents fibronectin staining, green represents α-actin staining, and blue represents MMP-14 staining. Scale bar represents 20 μm.

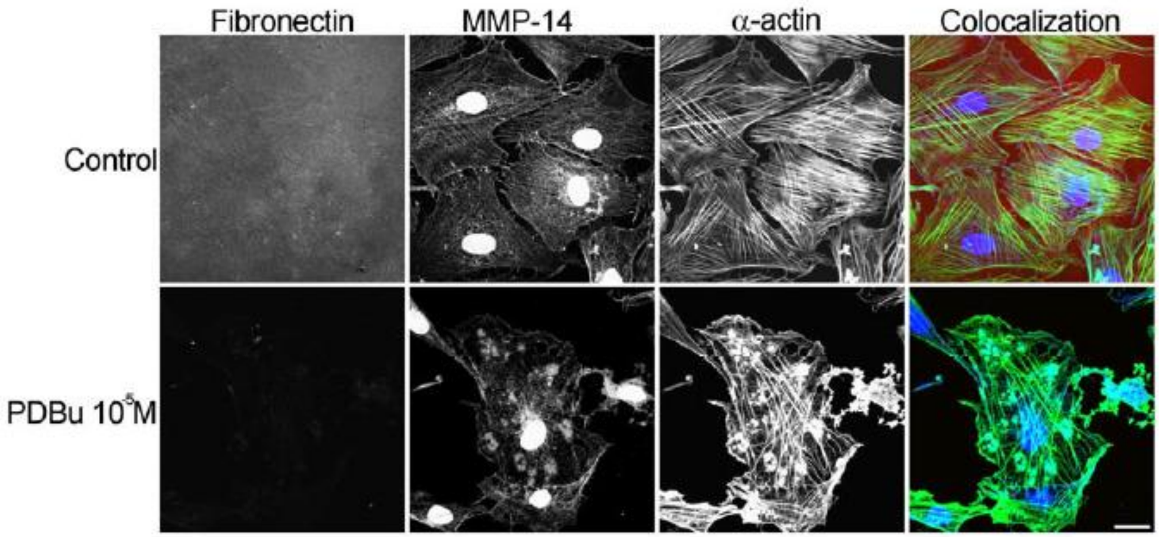

Supplemental Figure 3: Detection of protein carbonyls using the Oxyblot method. In **(a)**, Western blot of protein carbonyls and β-actin was used as a loading control. In **(b)**, quantitative summary of 3 independent experiments indicating an increase in oxidative stress under various concentrations of PDBu. In **(c)**, dihydroethidium (DHE) staining of A7r5 cells under control and PDBu-stimulated condtions (10^{-5} M). Superoxide accumulation was increased but not significant (P = 0.22).

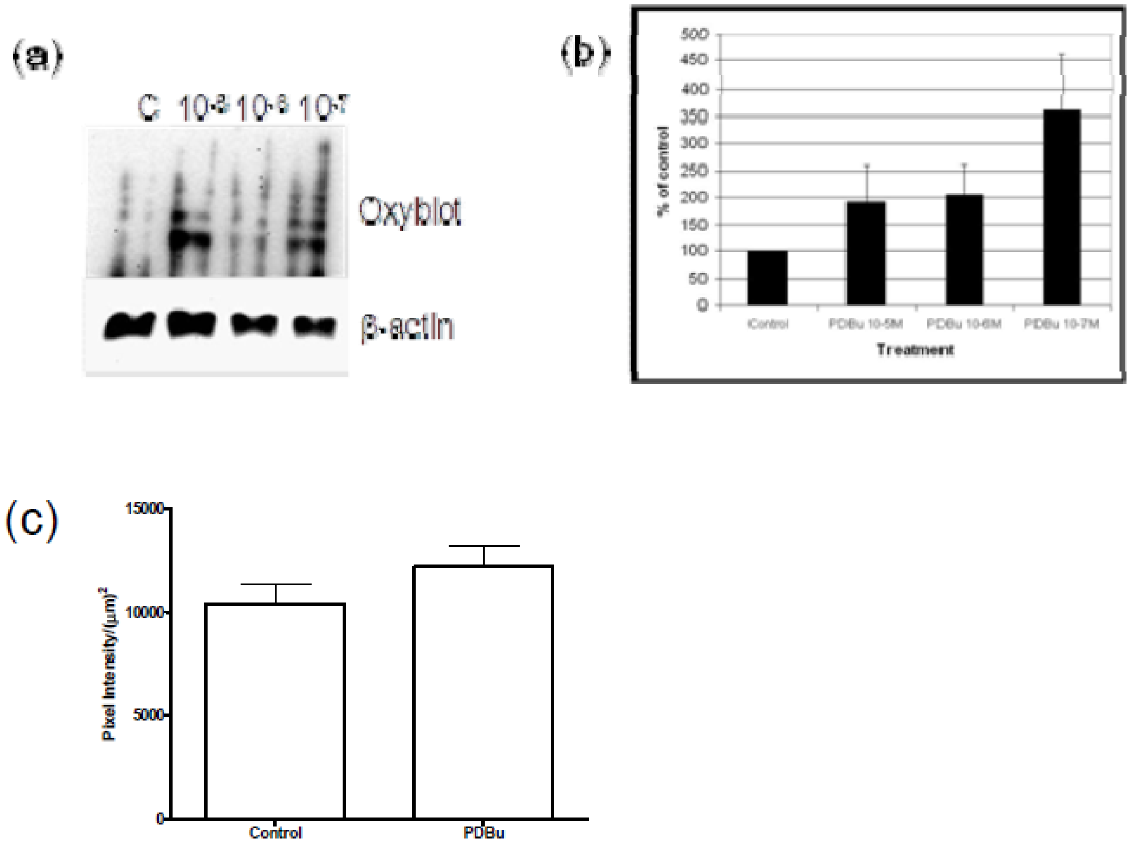

Supplemental Figure 4: (A) Colocalization of MMP-2 and paxillin in A7r5 cells and **(B)** slope analysis of pixel intensity of the two proteins in podosomes. Data indicates that localization of the two proteins were less than the MMP-9/α-actin interaction (m = 1.6). Ten different cells were examined and 13 different podosomes were examined under high magnification (1000X). Green represents MMP-2 staining and red represents paxillin staining under control and PDBu stimulating conditions (10^{-5} M). Scale bar represents 20 μm.

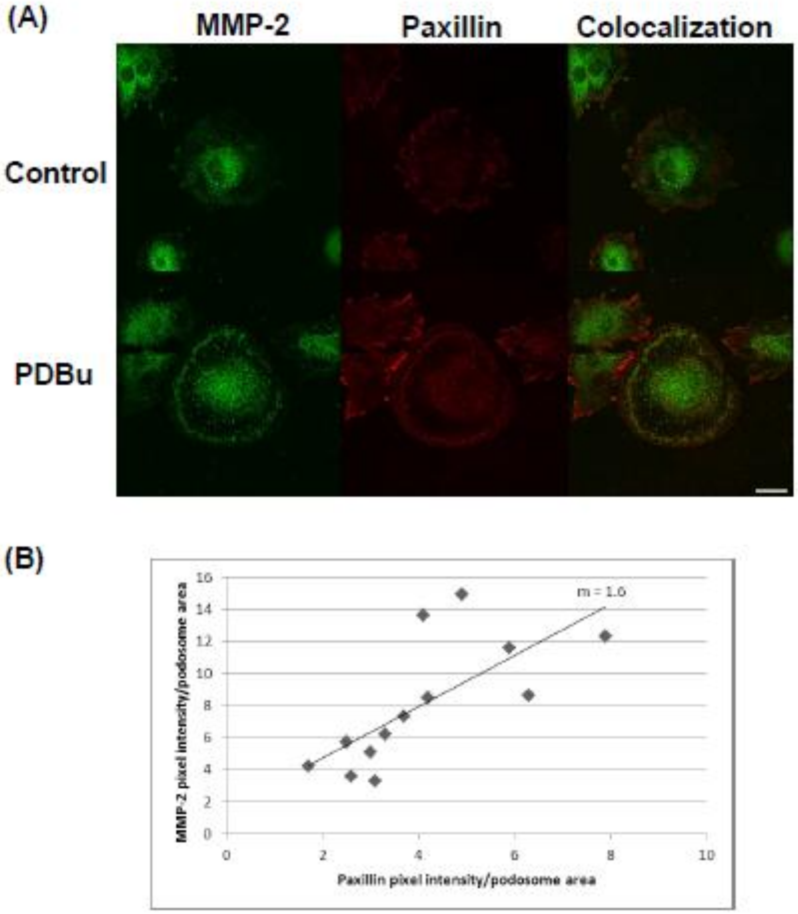

Supplemental Figure 5: Alpha-actin staining of A7r5 cells under PDBu- and pre-treatment of colchicine conditions. **(A)** Colchicine was given at 0.1 μM for 30 minutes before the addition of PDBu (10^{-5} M). **(B)** Percent of cells displaying podosomes were evaluated under both conditions and was not found to be significantly different. Scale bar represents 20 μm.

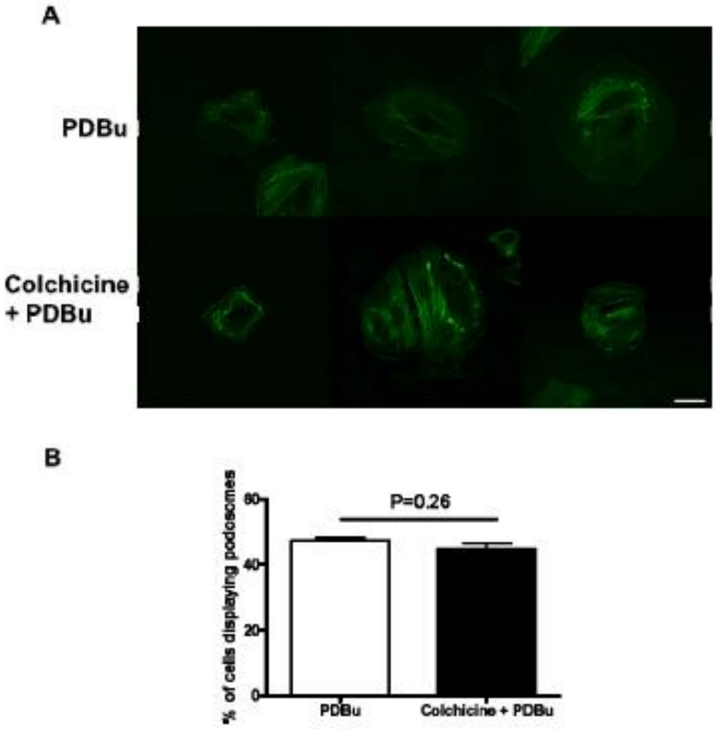

Supplement: JCB-2332-3671-05-0020 [file NIHMS877705-supplement-JCB-2332-3671-05-0020.pdf]
